# GMP-compliant fully automated radiosynthesis of [^18^F]FEPPA for PET/MRI imaging of regional brain TSPO expression

**DOI:** 10.1186/s13550-021-00768-9

**Published:** 2021-03-16

**Authors:** Chi-Wei Chang, Chuang-Hsin Chiu, Ming-Hsien Lin, Hung-Ming Wu, Tsung-Hsun Yu, Pao-Yeh Wang, Yu-Yeh Kuo, Ya-Yao Huang, Chyng-Yann Shiue, Wen-Sheng Huang, Skye Hsin-Hsien Yeh

**Affiliations:** 1 Department of Nuclear Medicine, Taipei Veterans General Hospital, Taipei, Taiwan; 2 Department of Medical Imaging and Radiological Technology, The Institute of Radiological Sciences, Tzu Chi University of Science and Technology, Hualien City, Taiwan; 3 Department of Biomedical Engineering and Environmental Sciences, National Tsinghua University, Hsinchu, Taiwan; 4 Department of Nuclear Medicine, Tri-Service General Hospital, Taipei, Taiwan; 5 Department of Nuclear Medicine, Cheng Hsin General Hospital, Taipei, Taiwan; 6 Department of Nuclear Medicine, Camillian Saint Mary’s Hospital Luodong, Yilan, Taiwan; 7 Department of Neurology, Changhua Christian Hospital, Changhua, Taiwan; 8 Brain Research Center, National Yang Ming Chiao Tung University, No. 155, Sec. 2, Linong Street, Taipei, 112 Taiwan; 9 PET Center, Department of Nuclear Medicine, National Taiwan University Hospital, Taipei, 100 Taiwan; 10 Molecular Imaging Center, National Taiwan University, Taipei, Taiwan; 11 PET Center, Department of Nuclear Medicine, Tri-Service General Hospital, Taipei, Taiwan; 12 Department of Nuclear Medicine, Taipei Medical University Hospital, Taipei, Taiwan

**Keywords:** [^18^F]FEPPA, Neuroinflammation, TSPO, Animal PET, MR imaging

## Abstract

**Background:**

Expression of translocator protein (TSPO) on the outer mitochondrial membrane of activated microglia is strongly associated with neuroinflammation. The second-generation PET ligand [^18^F]FEPPA specifically binds TSPO to enable in vivo visualization and quantification of neuroinflammation. We optimized a fully automated radiosynthesis method and evaluated the utility of [^18^F]FEPPA, the second-generation PET ligand specifically binds TSPO, in a mouse model of systemic LPS challenge to detect TSPO-associated signals of central and peripheral inflammation. In vivo dynamic PET/MR imaging was performed in LPS-induced and control mice after [^18^F]FEPPA administration. The relationship between the [^18^F]FEPPA signal and the dose of LPS was assessed. The cytokine levels (i.e., TNF-α, Il-1β, Il-6) in LPS-induced mice were measured by RT-PCR. Standard uptake value (SUV), total volume of distribution (VT) and area under the curve (AUC) were determined based on the metabolite-uncorrected plasma input function. Western blotting and immunostaining were used to measure TSPO expression in the brain.

**Results:**

The fully automated [^18^F]FEPPA radiosynthesis produced an uncorrected radiochemical yield of 30 ± 2% within 80 min, with a radiochemical purity greater than 99% and specific activity of 148.9‒216.8 GBq/µmol. Significant differences were observed in the brain after [^18^F]FEPPA administration: SUV, VT and AUC were 1.61 ± 0.1, 1.25 ± 0.12 and 1.58 ± 0.09-fold higher in LPS-injected mice than controls. TNF-α, Il-1β and Il-6 mRNA levels were also elevated in the brains of LPS-injected mice. Western blotting revealed TSPO (*p* < 0.05) and Iba-1 (*p* < 0.01) were upregulated in the brain after LPS administration. In LPS-injected mice, TSPO immunoactivity colocalized with Iba-1 in the cerebrum and TSPO was significantly overexpressed in the hippocampus and cerebellum. The peripheral organs (heart, lung) of LPS-injected mice had higher [^18^F]FEPPA signal-to-noise ratios than control mice.

**Conclusions:**

Based on the current data on ligand specificity and selectivity in central tissues using 7 T PET/MR imaging, we demonstrate that [^18^F]FEPPA accumulations significant increased in the specific brain regions of systemic LPS-induced neuroinflammation (5 mg/kg). Future investigations are needed to determine the sensitivity of [^18^F]FEPPA as a biomarker of neuroinflammation as well as the correlation between the PET signal intensity and the expression levels of TSPO.

**Supplementary Information:**

The online version contains supplementary material available at 10.1186/s13550-021-00768-9.

## Introduction

The 18-kDa mitochondrial translocator protein (TSPO), originally named peripheral-type benzodiazepine receptor (PBR), plays important roles in several physiological processes, including steroidogenesis [1, 2], inflammation [3] and cell proliferation [4]. TSPO is endogenously expressed at high levels in some peripheral tissues, including the adrenal gland, heart, lung, kidney and testis [3]. However, TSPO is expressed at very low levels in the central nervous system (CNS) under normal conditions, and limited to glial cells (astrocytes and microglia) [3, 5, 6].

Neuroinflammation is characterized by the activation of neuroimmune cells and implicated in the pathogenesis of several neurodegenerative diseases. Glial cells are activated in response to brain injury or neuroinflammation, and activated microglial/macrophages are associated with dramatically increased expression of TSPO [7]. High expression of TSPO in activated microglia has been reported in several neurodegenerative diseases, including Alzheimer’s disease [8], Huntington’s disease [9], ischemic stroke [10], multiple sclerosis [11] and epilepsy [12], which indicates TSPO plays essential roles in the progression of these diseases. Therefore, TSPO is considered to represent a relevant molecular marker for neuroinflammation and could possibly be an attractive therapeutic target. Thus, a specific biomarker for TSPO may improve early diagnosis and neuropathological follow-up after therapy, and even enable pharmacological screening of TSPO ligands.

Given the enhanced expression of TSPO in inflammatory cells in the injured brain, neuroimaging such as positron emission tomography (PET) or single-photon emission computed tomography (SPECT) employing TSPO radioligands may provide valuable approaches to track and quantify brain inflammation, and thereby assess the effectiveness of therapeutic interventions in real time. [^11^C]PK11195 was the first identified and is the most studied TSPO radioligand; however, due to its high lipophilicity, radiolabeled PK11195 exhibits high non-specific binding and leads to a poor signal-to-noise ratio, which complicates quantification [13]. Numerous second-generation TSPO-PET tracers have been developed to address these limitations, including [^11^C]CLINME [14], [^11^C]DAA1106 [15], [^11^C]SSR180575 [16, 17], [^18^F]FMDAA1106 [18, 19], [^18^F]PBR01 [20], [^18^F]PBR06 [21], [^18^F]PBR111 [22], [^11^C/^18^F]PBR28 [23], [^11^C]DPA-713 [24], [^18^F]DPA-714 [25], [^18^F]FEAC/FEDAC [26], [^18^F]FEPPA [27–30] and [^18^F]GE-180 [31].

Radiolabeled-TSPO ligands have been widely used to study the role of TSPO in animal models of chemical-induced neurotoxicity—such as lipopolysaccharide (LPS)-induced neurotoxicity—and also in models of human neurodegenerative disorders. These promising preclinical studies prompted clinical studies of TSPO ligands to detect neuroinflammation; however, these clinical studies produced mixed results. The first-generation radioligand [^11^C]PK-11195 was reported to lead to higher TSPO PET signals in the brains of patients with amyotrophic lateral sclerosis [32], Alzheimer’s disease [8], Parkinson’s disease [10] or individuals at risk of Huntington’s disease [33] compared to healthy controls. However, other TSPO PET studies using [^11^C]DAA1106 in cognitive impairment-dementia, [^18^F]FEDAA1106 in multiple sclerosis [19] and [^18^F]DPA-714 [34] or [^18^F]FEDAA1106 [35] in Alzheimer’s disease reported no significant differences. The second-generation TSPO ligand [^18^F]-*N*-(2-(2-fluoroethoxy)benzyl)-*N*-(4-phenoxypyridin-3-yl) acetamide ([^18^F]FEPPA) has superior properties to other ligands, including an 18-fold higher affinity than [^11^C]PK-11195 and metabolic stability in vivo [27, 36].

Administration of LPS—mainly by local intracerebral injection—is widely used to induce neuroinflammation in animals models for TSPO PET or neuroinflammation studies [37–39]. The metabolism and total volume distribution (VT) of [^18^F]FEPPA in the brain were previously determined in a murine model of systemic LPS-induced neuroinflammation [30].

However, even though the LPS-induced model is widely used, detection of regional-specific binding of TPSO in the brain using PET and the dynamic distribution of TSPO in the peripheral organs remain unclear in rodent LPS models. Furthermore, the effectiveness of [^18^F]FEPPA as a PET ligand in animals injected with varied doses of LPS requires further investigation. Moreover, an automated [^18^F]FEPPA radiosynthesis protocol that meets Good Manufacturing Practice is required for clinical purposes.

The overall aims of this study were to (1) optimize a fully automated radiosynthesis protocol for [^18^F]FEPPA on the Eckert-Ziegler modular system (Eckert-Ziegler, Berlin, Germany); (2) assess the regional-specific distribution of [^18^F]FEPPA in various brain regions and peripheral organs in mice with LPS-induced neuroinflammation; and (3) quantify the dose-dependence of the relationship between the dose of LPS and [^18^F]FEPPA PET signals in vivo.

## Materials and methods

### Optimized radiochemical synthesis of [^18^F]FEPPA

#### Reagents

All reagents and solvents were purchased from Huayi Isotopes Inc. (Suzhou, China) or Sigma-Aldrich (St. Louis, MO, USA) and used without further purification. Sep-Pak®Light QMA cartridges and tC18 plus Sep-Pak cartridges were obtained from Waters Corporation (Milford, MA, USA). The tosylated precursor of [18F]FEPPA, (2-(2-((*N*-4-phenoxypyridin-3-yl)acetamido)methyl)phenoxy)ethyl 4-methylbenzenesulfonate) and authentic FEPPA, N-(2-(2-fluoroethoxy)benzyl)-*N*-(4-phenoxypyridin-3-yl)acetamide were purchased from Huayi Isotopes Inc. [^18^F]fluoride ions were produced via the [18O(p,n)18F] nuclear reaction using a Scanditronix MC 17 cyclotron (Uppsala, Sweden). The radioactivity of the final product was measured with a dose calibrator (Capintec CRC-15R, Capintec, NJ, USA).

Radiosynthesis was performed using an Eckert-Ziegler modular system (Eckert-Ziegler, Berlin, Germany), and radiochemical yields were determined with a dose calibrator. Radioactive mixtures were purified by semi-preparative high-performance liquid chromatography (HPLC) using a Knauer pump P 2.1S (Knauer Corp., Berlin, Germany), Knauer UV/VIS detector UVD 2.1S (Knauer Corp.) and pin-diode radiodetector (Eckert-Ziegler). Quality control for the final product was performed by analytical HPLC using a Waters 2695 pump, Waters 2489 UV/VIS (254 nm) detector (Waters Corporation) and FC-4000 dual BGO coincidence detector (Bioscan, Inc., Washington, DC, USA). The amount of organic solvent was determined by gas chromatography (Varian Inc., Palo Alto, CA, USA).

#### Radiosynthesis

Radiosynthesis of [^18^F]FEPPA was performed using an Eckert-Ziegler modular system and a tosylated precursor via a one-step fluorine nucleophilic aliphatic substitution, based on a previously described radiosynthesis [27] (Additional file 2: Fig. S1). The reagents used in the automated in-house reaction sequence are listed in Additional file 1: Table S1.

The aqueous [^18^F]fluoride target solution was loaded onto a pre-conditioned QMA Sep-Pak® Light QMA cartridge (Waters Corporation). Concentrated [^18^F]fluoride was eluted into the reactor using 1.1 mL of a mixture of K_2_CO_3_ (2.75 mg) and Kryptofix (K222, 10.56 mg; CH3CN:H2O, 4:1, *v/v*). The solvents were evaporated under reduced pressure at 120 °C for 10 min. Five micrograms of the tosylated precursor (2-(2-((N-4-phenoxypyridin-3-yl)acetamido)methyl)phenoxy)ethyl 4-methylbenzenesulfonate) in acetonitrile (0.6 mL) were added to the dry residue containing the K222/potassium [^18^F]fluoride complex, and the mixture was heated to 90 °C for 10 min and cooled. The radiolabeling reaction was stopped by adding 2 mL of the mobile phase CH_3_CN:water (1:1 *v/v*) + 0.5% phosphoric acid into the reactor, and the solution was transferred into the injection loop. Purification was carried out by semi-preparative HPLC (column YMC-Actus Triart C18 250 × 20 mm I.D S-5 µm, 12 nm; Kyoto, Japan) in an isocratic (1:1 *v/v*) solution of CH3CN:water containing 0.5% phosphoric acid at a flow rate of 6 mL/min. The fraction containing [^18^F]FEPPA was collected at 13–14 min and mixed with 40 mL of water. [^18^F]FEPPA was subsequently trapped on a pre-conditioned (10 mL ethanol and 20 mL water) tC18plus SepPak cartridge, and the acetonitrile was washed off by rinsing the tC18plus SepPak cartridge with water. The product ([18F]FEPPA) was eluted with ethanol (0.9 mL) and formulated in normal saline (10 mL). The radiotracer solution was finally passed through a 0.22-µm Millipore filter into a sterile vial for in vivo experiments. The product was diluted in saline to obtain a volume activity of 2.2–2.8 GBq/mL.

#### Quality control

Radiochemical and chemical purity, stability and molar activity were assessed by analytical HPLC. The molar activity of the radiotracer was quantified by measuring the radioactivity injected and deriving the concentration of FEPPA in the sample by UV detection.

The identity of the labeled compound [^18^F]FEPPA was confirmed by co-injection with a non-radioactive FEPPA standard. The concentration of FEPPA in the radioactive sample was obtained from the UV-peak area ratio for the radioactive product and the standard solution. Analytical HPLC was performed on a Nova-pak C18 column (300 × 3.9 mm, 4 μm) in 50 mM (NH_4_)H_2_PO_4_:CH_3_CN (1:1) at 1 mL/min using a Waters Corporation 2695 pump with a multi-wavelength 2489 UV Detector (Waters Corporation) in series with a Bioscan FC-4000 dual BGO coincidence detector (Bioscan, Inc., Washington, DC, USA). A delay time of 12 s was observed between the two detectors.

### Animals

Eight-to-nine-week-old male C57BL/6 mice (23.64 ± 1.75 g) were housed with free access to water and maintained under controlled temperature (22 ± 2 °C) and humidity (55–65%) under a 12-h-light/dark cycle. All animal experiments were approved in advance by the National Yang-Ming University Institutional Animal Care and Use Committee (IACUC No: 1050910), and animal study was performed according to the Guidelines for Animal Experimentation of National Yang-Ming University.

### LPS-induced mouse model

The mice in the LPS group (6 mice for each dose) were intraperitoneally injected with a single dose of 0.625, 1.25, 2.5 or 5 mg/kg LPS (*Escherichia coli*, strain O111:B4; Calbiochem, San Diego, CA, USA) to induce systemic inflammation. The control group were intraperitoneally injected with 0.9% NaCl. In vivo or in vitro assessments were performed 24 h after administration of LPS.

### In vivo PET/MRI imaging and imaging analysis

Before PET/MR imaging acquisition, the mice were anesthetized by passive inhalation of a mixture of isoflurane and oxygen (5% isoflurane for induction and 2% for maintenance). A fiber-optic temperature probe was inserted into the rectum to monitor the core temperature of the mice, then each animal was gently positioned in the MR cradle and the MRI coil was secured around the head. A warm water blanket was positioned to maintain the core temperature of the animals, then the mice were positioned inside the scanner. The depth of anesthesia, pulse and respiration were monitored constantly during the imaging procedure; in the unlikely event that an animal regained consciousness, the scanning was immediately stopped and the animal was removed from the scanner, humanely euthanized and eliminated from the study.

The animals (*n* = 6 per dose of LPS; *n* = 6 controls) were injected with [^18^F]FEPPA (10.09 ± 1.38 MBq; 0.31 ± 0.02 mCi) via the tail vein. Dynamic PET images were obtained using a 7 T PETMR Inline (Bruker, Rheinstetten, Germany) for 30 min with the energy window set to 350–650 keV. Images were acquired every 1 s for 10 images, 10 s for five images, 60 s for nine images, 300 s for two images and 600 s for 1 image; a total of 27 frames were collected.

T1 and T2 MRI were performed to determine the anatomical structure of the brain. The MRI sequences included 0.5-mm thickness coronal T2 Turbo RARE high-resolution images (TR = 3455 ms, TE = 36 ms, matrix = 256 × 256, average = 8, slice number = 30).

The PET images were reconstructed through three-dimensional ordered-subset expectation maximization (3D-OSEM). The regional radioactivity concentration (KBq/cc) of [^18^F]FEPPA was estimated based on the mean pixel values within the volumes of interest (VOI) corresponding to MR images of various regions of the brain. Image data were decay-corrected to the injection time. The radioactivity concentration (KBq/cc) of the VOI was converted to the standard uptake value (SUV), and the mean and standard error of the mean (SEM) radiotracer accumulation values were calculated for various tissues. PET/MR data were analyzed using PMOD 4.0 software (PMOD Technologies Ltd., Zurich, Switzerland).

### Quantification of dynamic PET imaging using logan graphical analyses

The Logan graphical method for reversible uptake [40] was used to assess whether [^18^F]FEPPA PET/MRI [30] could be used to detect differences in the expression of TSPO. Cardiac blood was used as the reference tissue. The slope of the linear portion of the Logan plot represents the total distribution (VT). If metabolite-corrected plasma TACs are not available, a reference region TAC, *C*_p_(*t*), can be used instead of the plasma TAC. Then, the slope of the linear portion of the plot can be calculated using (Eq. 1)

1 $$\frac{{\int\limits_{0}^{t} {C_{{{\text{Tissue}}}} \left( \tau \right){\text{d}}r} }}{{C_{{{\text{Tissue}}}} \left( t \right)}} = K\frac{{\int\limits_{0}^{t} {C_{{\text{p}}} \left( \tau \right){\text{d}}r} }}{{C_{{{\text{Tissue}}}} \left( t \right)}} + V$$

Logan graphical analyses were performed using PMOD 4.0 software.

### Real-time reverse transcription-polymerase chain reaction

To assess the transcript levels of the genes encoding the cytokines TNF-α, Il-1β and Il-6 in the brain following LPS-induced systemic inflammation, total RNA was isolated from the brains of the mice after imaging at the indicated time points after LPS injection. RNA was isolated using the RNeasy Micro Kit (Qiagen, Valencia, CA, USA) and cDNA was prepared using the Super Script III First-Strand Synthesis System Kit (Invitrogen, Carlsbad, CA, USA). RT-qPCR was performed for a total of 40 cycles using a Rotor-Gene Q cycler (Qiagen, Hilden, Germany), according to the manufacturer’s protocol using TaqMan gene expression master mix, primers and MGB probe sets (Applied Biosystems, Foster City, CA, USA). The assay IDs for the PCR primer pairs and TaqMan MGB probes were Mm00443260_gl (*Tnf-α*), Mm00434228_ml (*Il-1β*), Mm00446190_ml (*Il-6*) and Mm00607939Msl (*β-actin*). Relative expression levels were calculated using the comparative threshold cycle (Ct value) method and normalized to the ΔCt of *β-actin*. All analyses were conducted in triplicate.

### Western blotting

After imaging, brain tissues were lysed in ice-cold radioimmunoprecipitation assay buffer and total protein lysates were fractionated by sodium dodecyl sulfate–polyacrylamide gel electrophoresis and transferred to polyvinylidene difluoride membranes. The membranes were probed with antibodies against iba-1 (GTX100042) from GeneTex Biotechnology (Irvine, CA) at 1/1000 dilution, and TSPO (NB100-41398) from Novus Biologicals (Centennial, CO, USA) at 1/10,000 dilution and β-actin (NB600-501) from Novus Biologicals (Centennial, CO, USA) at 1/1000 dilution. After washing three times for 10 min in Tris-buffered saline supplemented with 0.1% Tween 20 (TBST), the membranes were incubated with horseradish peroxidase-conjugated secondary antibodies for 1 h, washed with TBST and the signals were visualized and quantified using the GeneGnome Chemiluminescence Imaging System (Syngene, Frederick, MD, USA).

### Immunofluorescence and immunohistochemistry

For immunofluorescence staining, after imaging, the mice were terminally anesthetized, perfused with 4% paraformaldehyde, the brains were dissected, post-fixed overnight in 4% paraformaldehyde at 4 °C, embedded in optimal cutting temperature compound and frozen coronal sections (30-μm thick) were prepared. The sections were permeabilized with 1% Triton X-100 in phosphate-buffered saline (PBS) for 10 min, blocked with 1% bovine serum albumin in PBS-T for 1 h, incubated with antibodies against Iba-1 (GTX100042) from GeneTex Biotechnology (Irvine, CA) at 1/1000 dilution and TSPO (NB100-41398) from Novus Biologicals (Centennial, CO, USA) at 1/10,000 dilution. The sections were incubated with 3′-diaminobenzidine for 20 s or fluorescein isothiocyanate-conjugated donkey anti-rabbit antibody (1:200) at room temperature for 1 h and counterstained with 4′,6-diamidino-2-phenylindole for 5 min at room temperature.

For immunohistochemistry staining, paraffin-embedded sections were cut at 5-µm thickness, deparaffinized and rehydrated. Antigen retrieval was carried out by microwaving in 10 mM citrate buffer (pH 6.0) at 100 °C for 10 min. Sections were washed, incubated in 3% hydrogen peroxide for 15 min at room temperature, incubated in blocking solution for 60 min at room temperature and incubated overnight with TSPO primary antibody (#STJ11 2662, 1:50; St John's Laboratory Ltd., London, UK) at 4 °C. Biotinylated secondary antibodies and 3,3-diaminobenzidine from the Vectastain Elite Kit (PK-6100, Vector Laboratories, Burlingame, CA, USA) were applied according to the manufacturer’s protocol.

Immunostained sections were assessed using an AxioScope A1 microscope (Zeiss, Oberkochen, Germany) equipped with a Zeiss Axiocam 512 color digital camera. The IHC images were converted into 8-bit grayscale images in the range [0–255]. The regions of interest (ROI) were manually selected and the intensity of immunostaining was measured using the software. The median (25%, 75% interquartile range) percentage score for each group was calculated as the average of all mice in each group.

### Statistical analysis

Data are expressed as mean ± SEM. One-way ANOVA with the post-hoc Bonferroni or unpaired Student’s *t*-test were used for statistical evaluation. *P* < 0.05 was considered statistically significant. Statistical analyses were performed using GraphPad Prism 8 (GraphPad Software, La Jolla, CA, USA).

## Results

### Automated radiosynthesis of [^18^F]FEPPA

Fully automated radiosynthesis of [^18^F]FEPPA was achieved within 80 min. The overall production of [^18^F]FEPPA was 30 ± 2% (decay-uncorrected, *n* = 8). Specific activity at the end of synthesis ranged from 148.9 to 216.8 GBq/µmol (*n* = 8).

### Quality control and stability tests of [^18^F]FEPPA

**Fig. 1:**
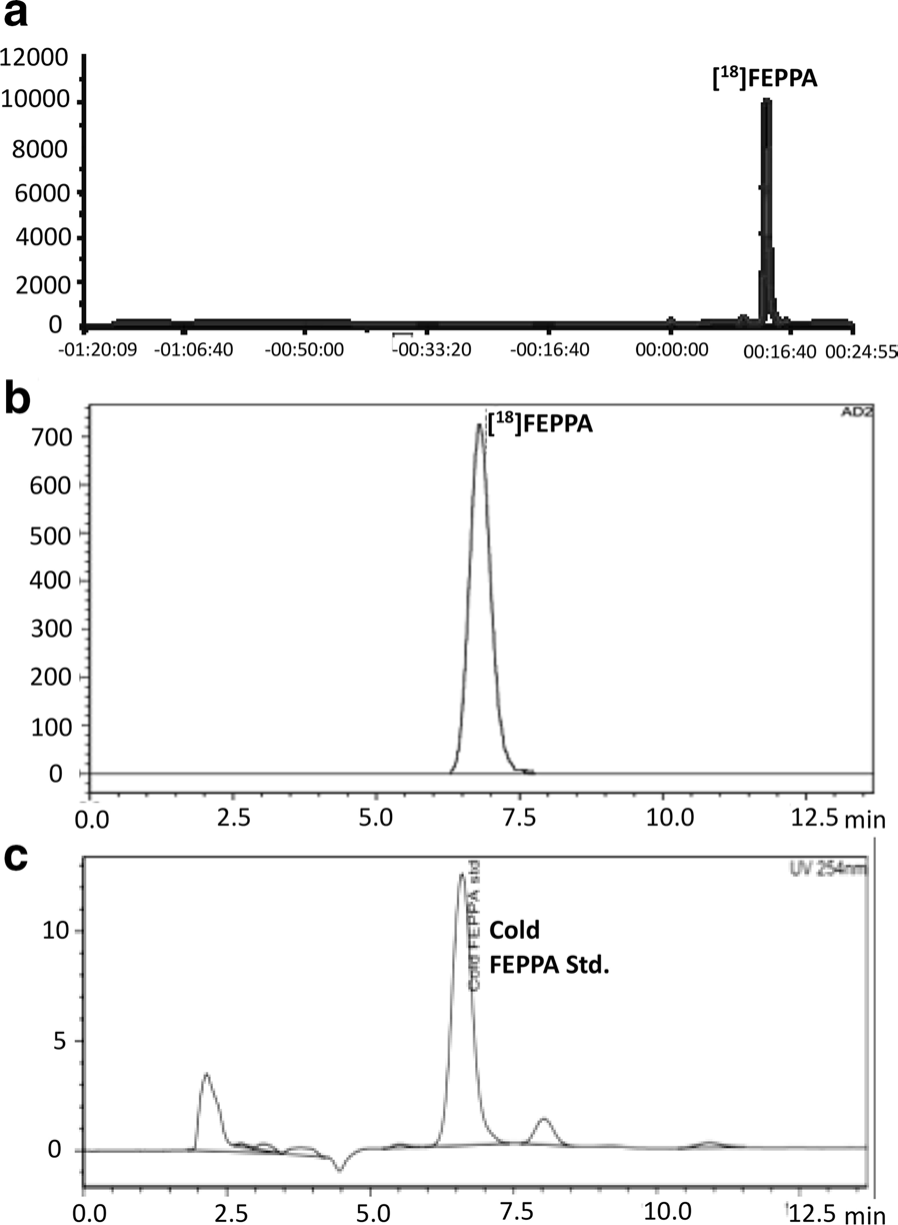
**a** The retention time (Rt) of [^18^F]FEPPA in semi-preparative HPLC analysis was 13.07 min. **b** The retention time (Rt) of [^18^F]FEPPA in HPLC analysis was 6.8 min. **c** The retention time (Rt) of authentic FEPPA in HPLC analysis was 6.6 min

The retention time (Rt) of [^18^F]FEPPA in semi-preparative HPLC was 13.07 min (Fig. 1a, radio-peak). The radioactive product was also co-injected with an authentic FEPPA standard. The retention time (Rt) of [^18^F]FEPPA (Fig. 1b, radio-peak) in HPLC analysis was 6.8 min, which was consistent with that of authentic FEPPA (Fig. 1c, UV peak).

One typical production batch of [^18^F]FEPPA was assessed using all of the quality control tests required for human use. In this production, the levels of residual solvents and K2.2.2 were below the levels set by the Taiwan FDA and the values for all other tests all met the specifications for human use.

### Quantitative whole-body biodistribution in mice

**Fig. 2:**
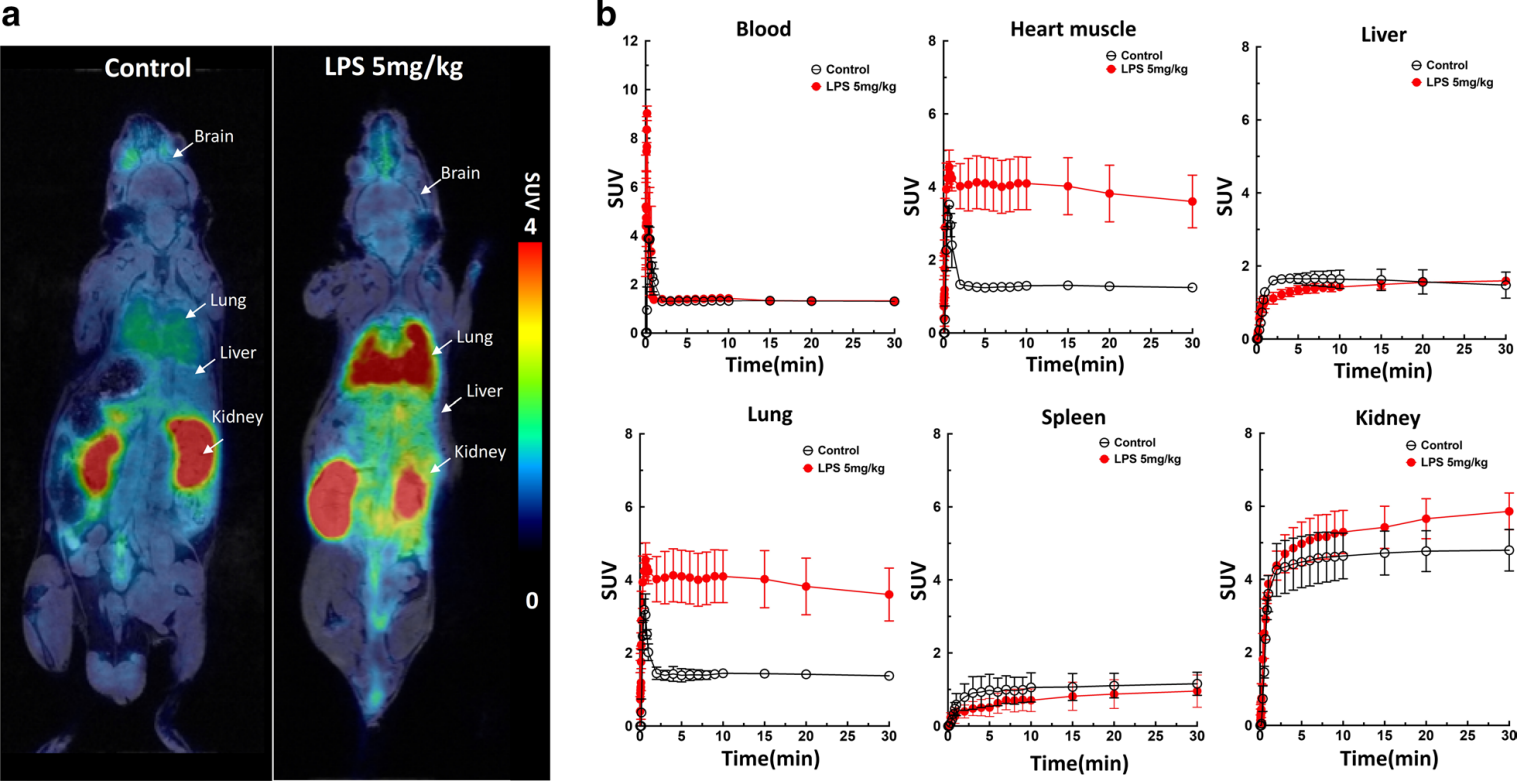
**a** Representative whole-body coronal PET/MR images of [^18^F]FEPPA in control and 24h LPS-injected animals (5 mg/kg, i.p.). PET images were generated by summation of the whole scan (0–30 min) and color-coded to the range of SUV values. **b** Time activity curves for [^18^F]FEPPA radioactivity (SUV) in blood, heart muscle, liver, lung, spleen and kidney. Data are mean ± SEM of 6 mice per group

In vivo dynamic PET/MR imaging was performed in six mice at 24 h pretreated with 5 mg/kg LPS and six control mice after administration of [^18^F]FEPPA. Generally, PET/MR imaging revealed rapid accumulation of [^18^F]FEPPA in LPS-injected mice, and—to a lesser degree—in the control group (Fig. 2a). In the control group, [^18^F]FEPPA exhibited biexponential blood clearance kinetics after *i.v.* injection and the concentration of radioactivity remaining in the blood pool was 1.29 ± 0.03 SUV at 30 min post-injection (Fig. 2b). The radioactivity levels in liver indicated rapid uptake within 0–1 min post-injection (Additional file 3: Fig. S2), and slightly increasing at later time points whereas progressively increasing kidney radioactivity during the 3–30 min predominantly represents the renal clearance of [^18^F]FEPPA. Rapid accumulation of [^18^F]FEPPA in the lungs, peaking at 1–2 min post-injection, was also observed (Additional file 3: Fig. S2). The concentration of radioactivity in heart muscle peaked in the first 3 min after injection, and then gradually decreased over time (Fig. 2b and Additional file 3: Fig. S2). Detailed results of the biodistribution of [^18^F]FEPPA in vivo are listed in Table 1.

**Table 1:** Biodistribution of accumulated radioactivity (SUV) in various tissues at the designed time points after administration of [^18^F]FEPPA

Tissues	Control	LPS (5 mg/kg)
Blood	1.29 ± 0.03	1.29 ± 0.128
Heart muscle	1.24 ± 0.06	3.60 ± 0.722*
Liver	1.48 ± 0.36	1.60 ± 0.097
Lung	1.38 ± 0.08	3.60 ± 0.722**
Spleen	1.16 ± 0.32	0.96 ± 0.443
Kidney	4.80 ± 0.57	5.86 ± 0.504
Whole brain	0.48 ± 0.05	0.76 ± 0.014

Data are mean ± SEM

*p < 0.05; **p < 0.01, One-way ANOVA with Bonferroni post hoc test

### In vivo brain region-specific uptake of [^18^F]FEPPA

**Fig. 3:**
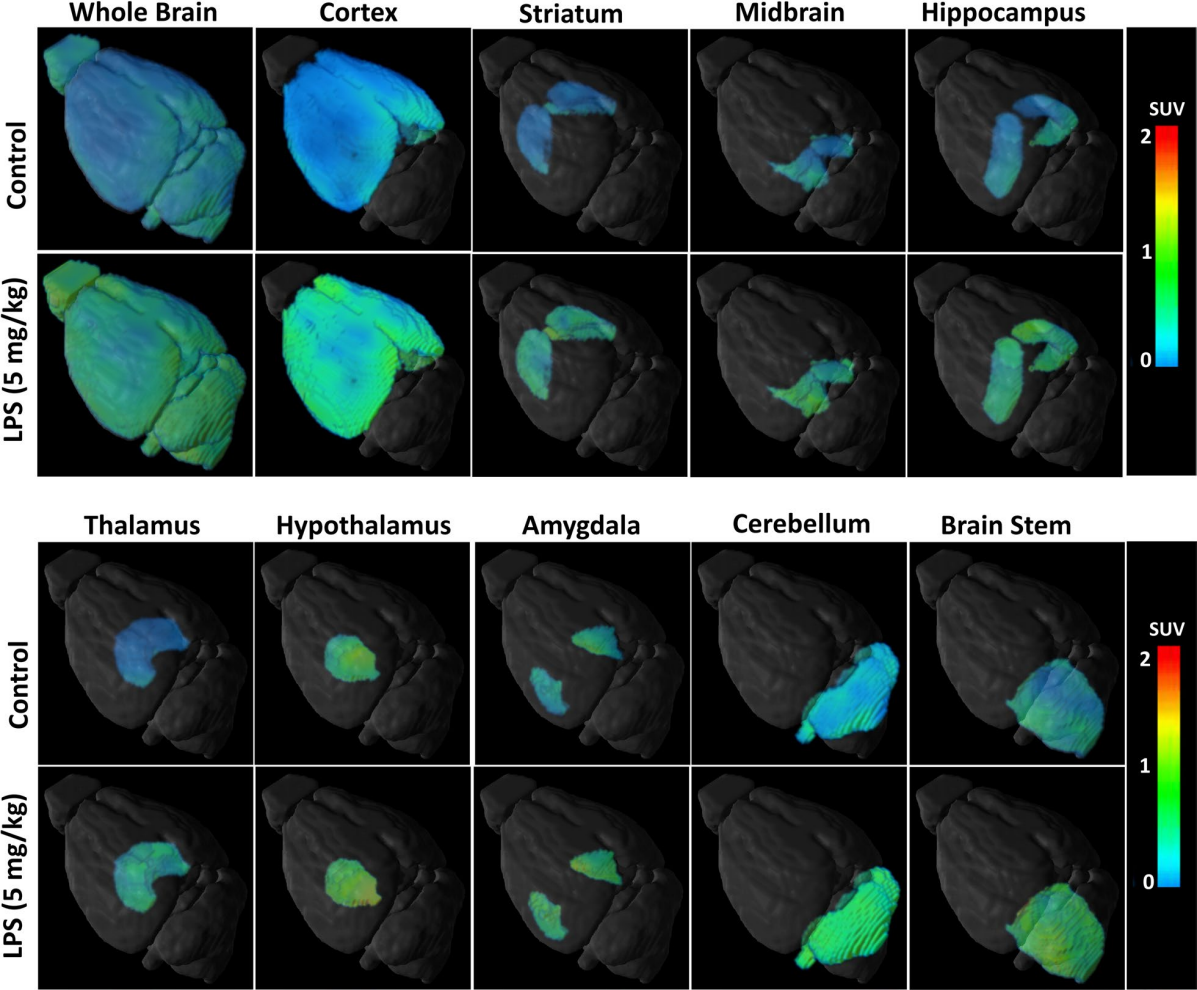
3D PET/MRI images of the brain obtained 30 min after *i.v.* administration of [^18^F]FEPPA in control and 24 h LPS-injected animals (5 mg/kg, i.p.). Significantly higher [^18^F]FEPPA uptake was observed in all brain regions of the LPS group at 24 h compared to the control group. [^18^F]FEPPA TSPO binding were more region-specific or globally distributed in the brain. PET images were generated by summation of the whole scan (0–30 min); the mean SUV value of each group is represented and color-coded to the range of SUV values (*n* = 6–8 mice per group)

**Fig. 4:**
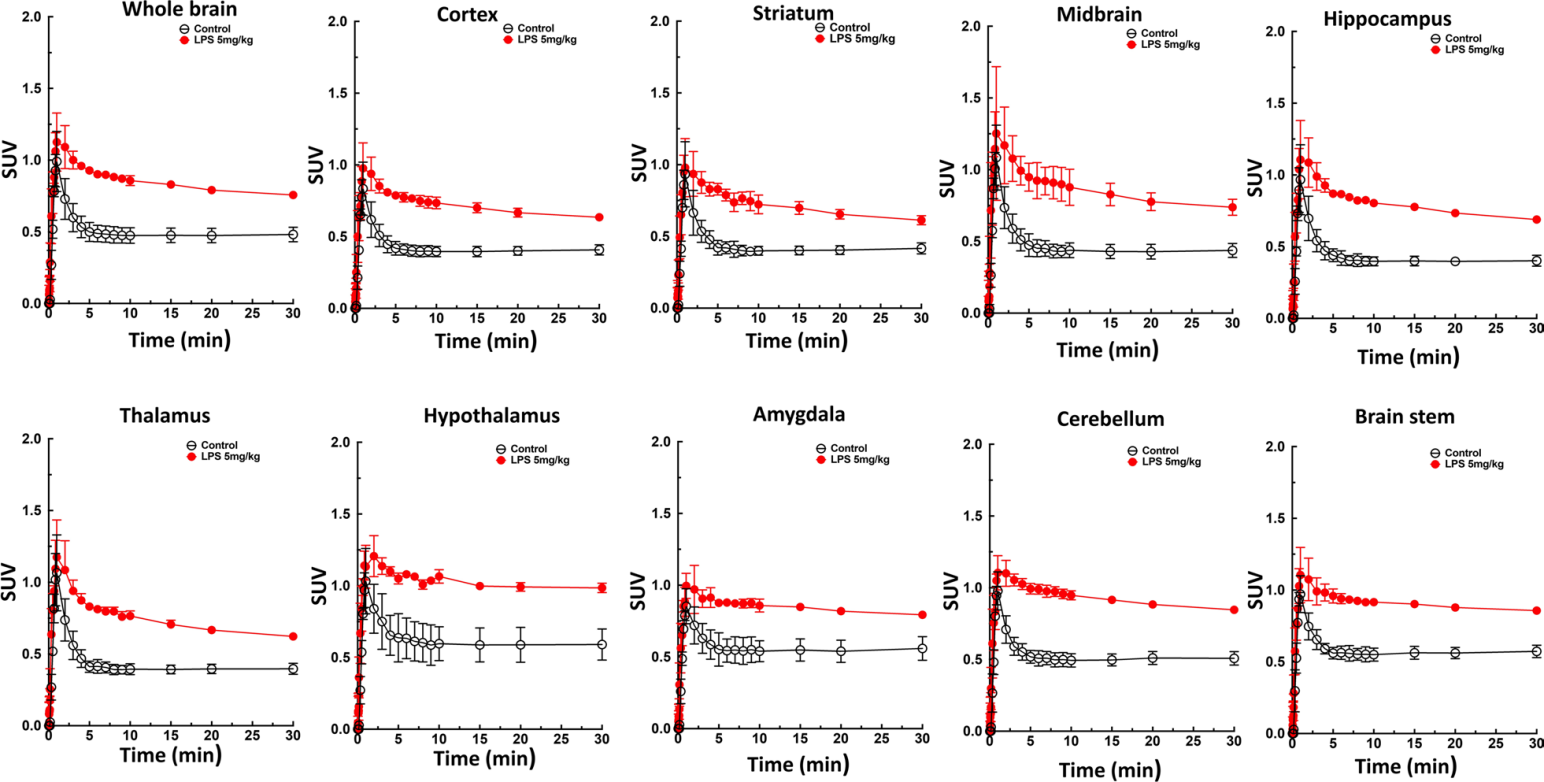
Time-activity curves for animal PET/MR studies of [^18^F]FEPPA of the brain for the control group and 24 h after LPS injection (5 mg/kg). Significantly higher [^18^F]FEPPA uptake was observed in all brain regions of the LPS group at 24 h compared to the control group. Data are mean ± SEM of 6–8 mice per group

The 3D PET/MRI brain images shown in Fig. 3 illustrate the [^18^F]FEPPA signals in the control group and LPS group at 24 h after LPS challenge. As shown in Fig. 4, significantly higher [^18^F]FEPPA uptake was observed in all brain regions of the LPS group at 24 h compared to the control group. The highest uptake was detected in the hypothalamus, followed by the midbrain, hippocampus, thalamus, brain stem, whole brain, cortex, striatum, cerebellum and amygdala. In control animals, the regional [^18^F]FEPPA time-activity curves indicated radioactivity peaked during the first 3 min, followed by rapid washout (Additional file 4: Fig. S3). In contrast, [^18^F]FEPPA accumulated more rapidly in the brain regions of the LPS group in the first 1 min and then continued to gradually wash out from these regions (Additional file 4: Fig. S3).

### Pharmacokinetic characterization of [^18^F]FEPPA in the brains of LPS-induced mice

**Fig. 5:**
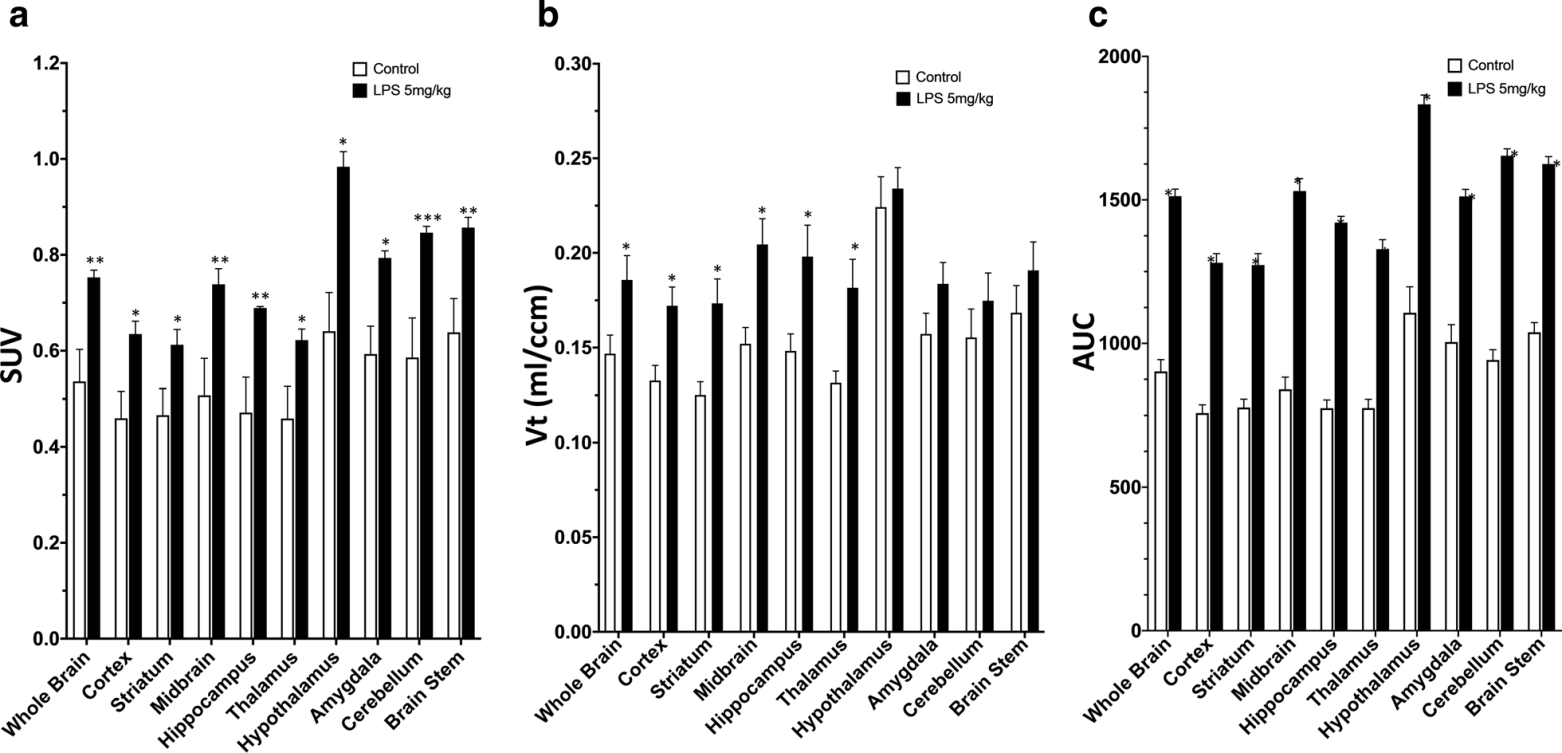
Pharmacokinetics of [^18^F]FEPPA after *i.v.* administration. Quantitative analysis of radioactivity in control mice and LPS-injected mice (5 mg/kg). **a** SUV, **b** VT, and **c** AUC. Data are mean ± SEM of 6–8 mice per group; *p < 0.05, **p < 0.01

The brain regions of the LPS group exhibited significantly higher SUV, VT and AUC values compared to the control mice. Generally, the [^18^F]FEPPA SUV were significantly different between control and LPS animals from the first 5 min onwards, and the average SUV ratio for all brain regions between the two groups was 1.61 ± 0.1 (Fig. 5a).

PET images were quantified using Logan graphical analysis. The VT values for [^18^F]FEPPA PET in the majority of brain regions were significantly higher in the LPS group than the controls, except for the hypothalamus, brain stem and cerebellum. On average, the [^18^F]FEPPA VT values were 1.25 ± 0.12-fold higher in the LPS group than the control group (Fig. 5b).

Integrated activity (AUC; SUV·min, 0–30 min) revealed rapid cerebral distribution and higher retention of [^18^F]FEPPA in the LPS group (Fig. 5c). The AUC ratio between the LPS and control group was 1.58 ± 0.09. Significantly higher [^18^F]FEPPA AUCs are consistent with overexpression of the target in the brain of mice injected with LPS, reflecting the well-characterized ability of LPS to induce inflammation and upregulate TSPO.

### Quantitative analysis of LPS dose response and brain distribution

**Figure. 6:**
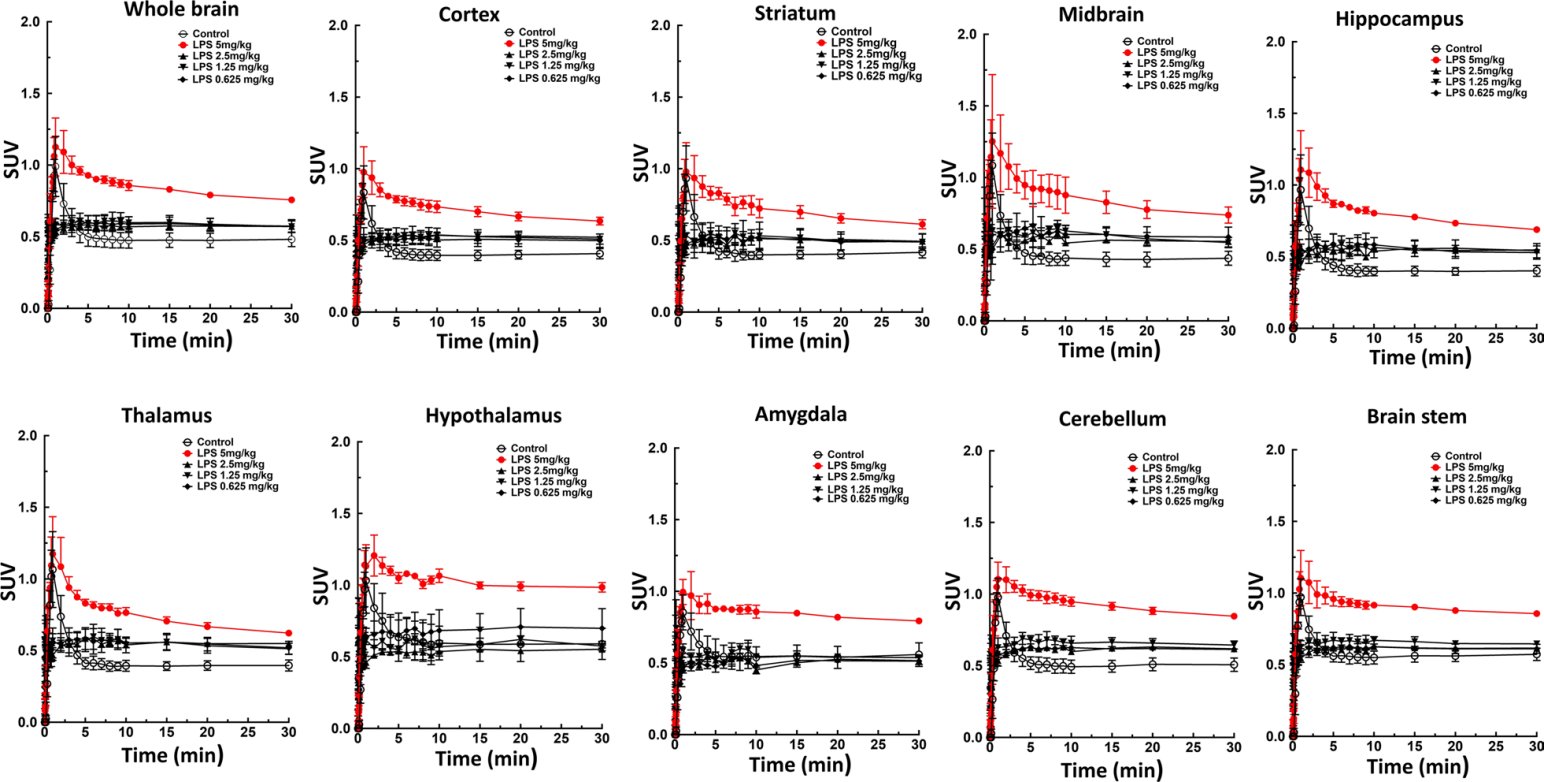
Time-activity curves for [^18^F]FEPPA in different regions of the brain based on PET/MRI imaging 24 h after injection of various doses of LPS. There was no significant difference in brain [^18^F]FEPPA uptake when the dose of LPS was ≦ 2.5 mg/kg, but significantly higher uptake was observed at 5 mg/kg LPS. Data are mean ± SEM of 6–8 mice per group

**Fig. 7:**
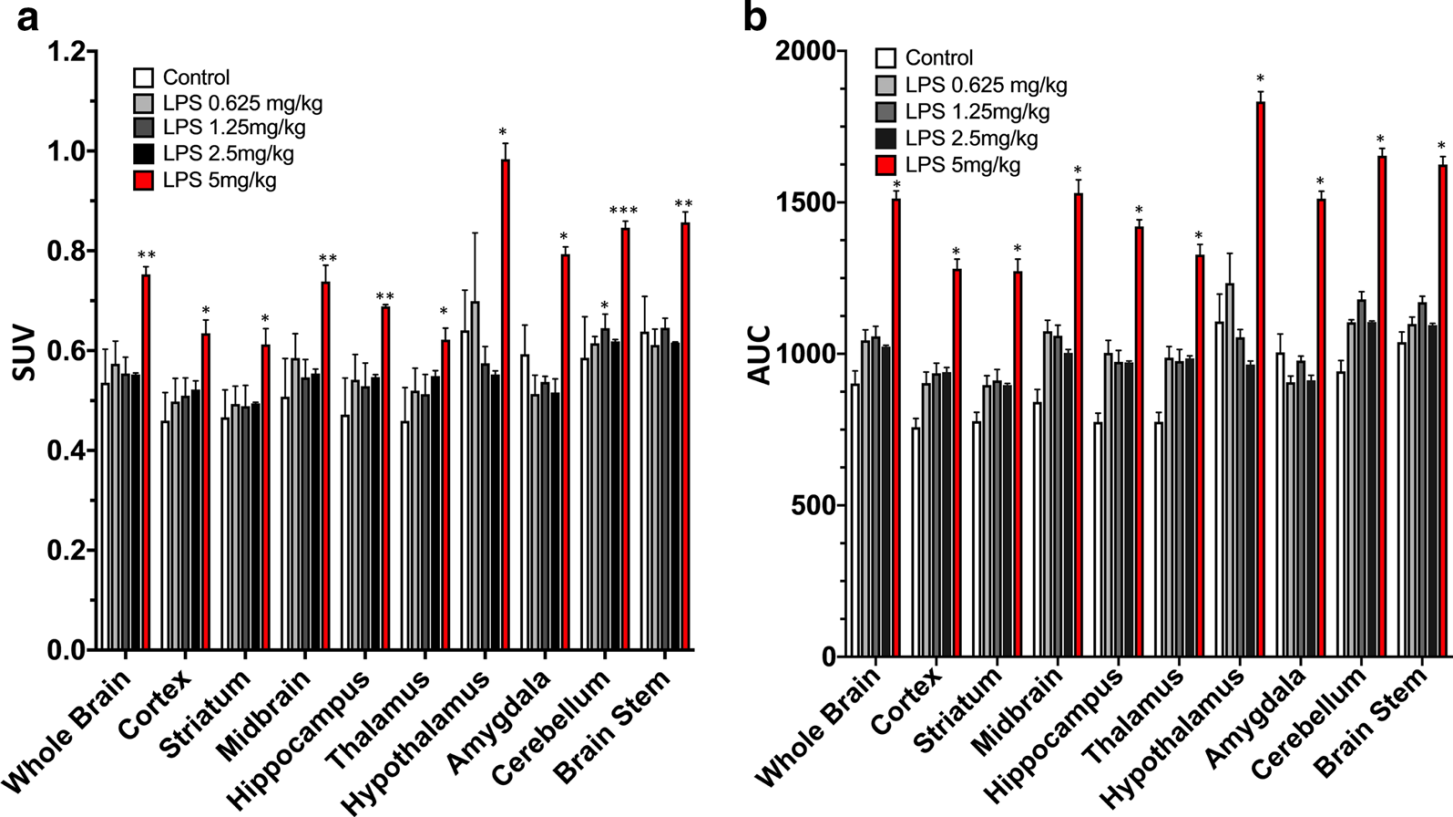
[^18^F]FEPPA SUV and AUC for various brain regions in control mice and mice injected with different of doses LPS. Data are mean ± SEM of 6–8 mice per group; *p < 0.05, **p < 0.01, **p < 0.001, one-way ANOVA with Bonferroni post hoc test

To assess whether the [^18^F]FEPPA uptake dose–response correlates with LPS exposure, we quantified [^18^F]FEPPA uptake in various brain regions after administration of different doses of LPS. The time-activity curves for various brain regions after injection of LPS are presented in Fig. 6. There was no significant correlation between [^18^F]FEPPA SUV uptake or AUC and the dose of LPS at concentrations up to 2.5 mg/kg; though the SUV values significantly increased at 5 mg/kg LPS (Fig. 7a, b).

### LPS stimulates proinflammatory cytokine secretion

**Fig. 8:**
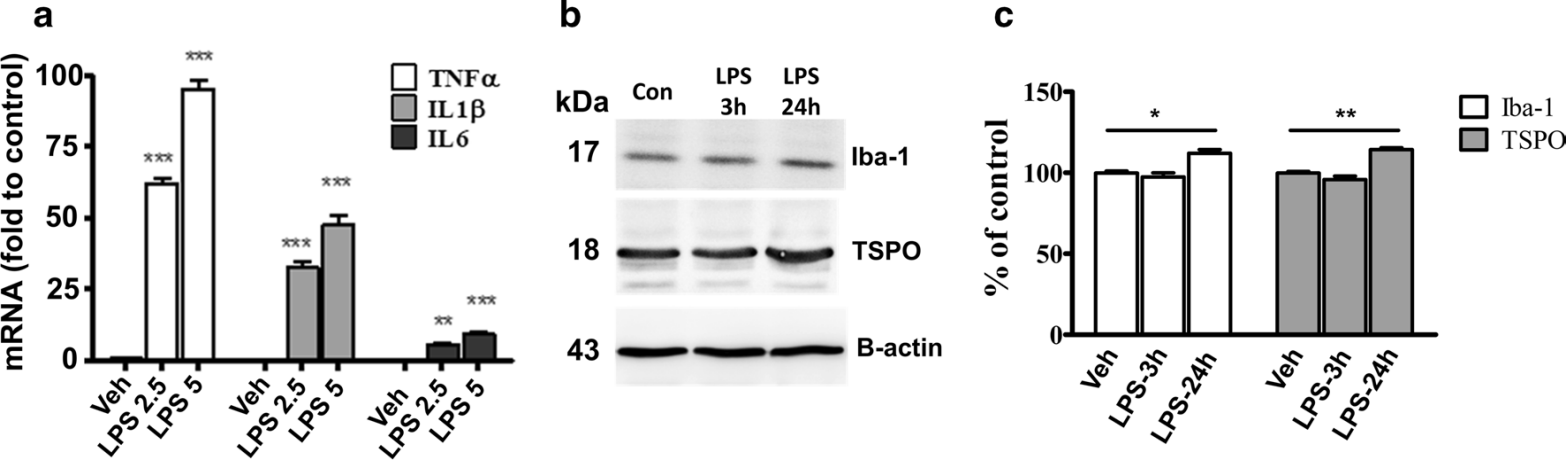
**a** Cytokine mRNA expression levels of TNF-α, Il-1β and Il-6 in the brain tissues 24 h after LPS injection. **b** the levels of Iba-1 and TSPO at 3 and 24 h after injection of 5 mg/kg LPS determined by immunoblots, **c** Quantitative analysis of protein levels of immunoblots. Data are mean ± SEM (*n* = 3 mice per group); *p < 0.05, **p < 0.01, ***p < 0.005

To investigate the proinflammatory reaction induced by LPS, we measured the mRNA expression levels of the proinflammatory cytokines TNF-α, IL-1β and IL-6 in brain homogenates at 24 h after injection of LPS. As shown in Fig. 8A, the mRNAs encoding TNF-α and IL-1β were expressed at higher levels in the brain homogenates of the LPS-group than the control group (LPS 2.5 mg/kg vs. control, *P* < 0.01; LPS 5 mg/kg vs. control,* P* < 0.001), demonstrating that injection of LPS-induced inflammation and the release of proinflammatory cytokines. Thus, we next examined the dose-dependence of cytokine expression and release.

### Upregulation of TSPO expression after LPS induction in mice

**Fig. 9:**
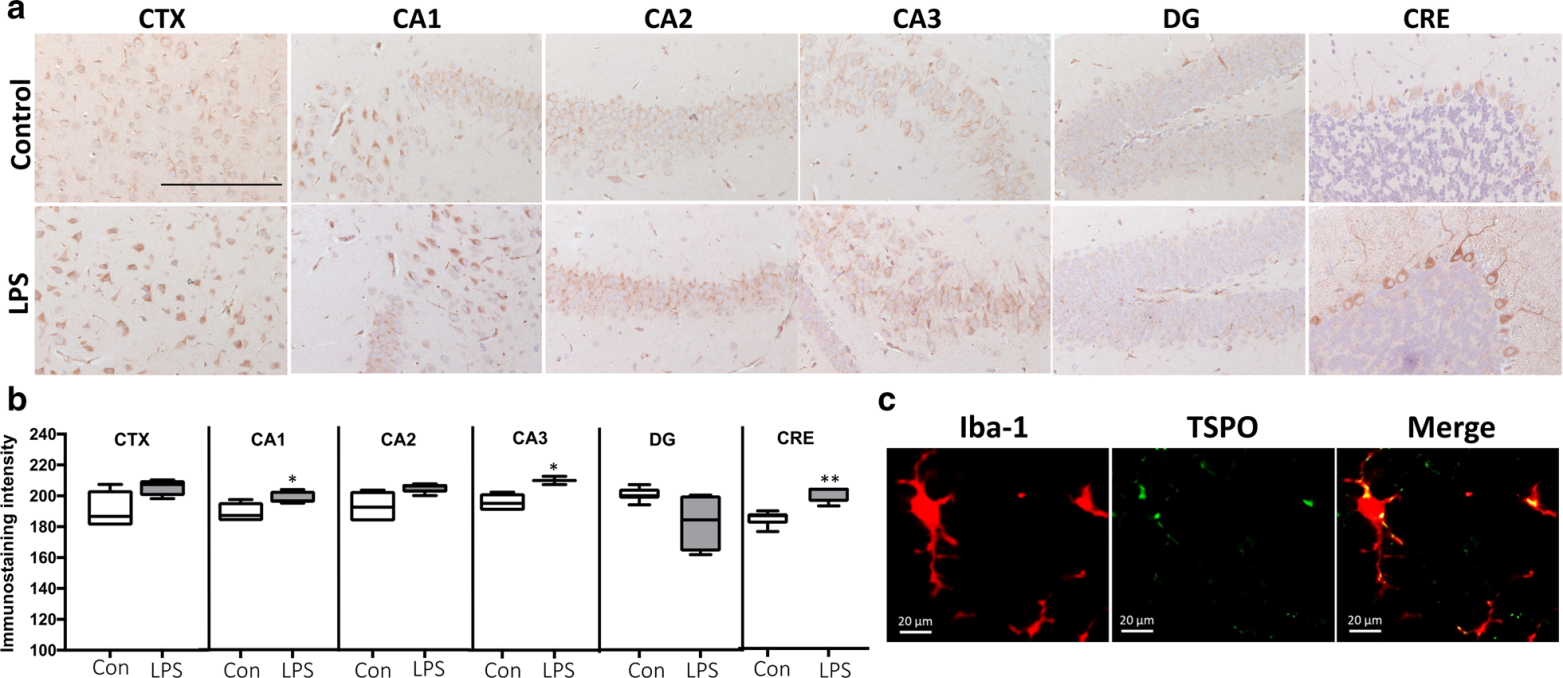
**a** Immunostaining of the cortex, CA1, CA2, CA3, DG and cerebellum for TSPO. Scale bar: 100 µm. **b** Intensity of TSPO immunostaining in six selected brain regions of control and LPS-injected mice: CTX-Cortex, CA1, CA2, CA3, DG—hippocampus, CRE-cerebellum. Data are mean ± SEM (*n* = 3 mice per group). **c** Expression of TSPO (green) colocalized with the microglia marker Iba-1 (red) (*n* = 3 mice per group). Scale bar: 20 μm. *p < *0.05*, **p < 0.01

The TSPO band was detected at 18 kDa in Western blotting (Fig. 9b). A marked increase in TSPO expression was observed in the brain 24 h after administration of LPS (*P* < 0.05); Iba-1 expression also increased in the LPS group at this time point (*P* < 0.01; Fig. 9c). Western blotting revealed time-dependent induction of TSPO expression, in confirmation of the PET/MR imaging studies.

### Quantitative immunostaining confirms the results of in vivo PET/CT imaging with [^18^F]FEPPA

TSPO activity was also analyzed in the brain cortex, hippocampus and cerebellum at 24 h after LPS injection (Fig. 9a). TSPO immunoreactivity was significantly higher in the CA1, CA3 and cerebellum at 24 h after administration of LPS, whereas TSPO expression was similar in the CA2 and dentate gyrus between the control and LPS groups. A tendency toward higher expression of TSPO was observed in the cortex of LPS mice; however, this trend was not significant (Fig. 9b).

Cerebral expression of TSPO colocalized with the microglial marker Iba-1 in mice injected with LPS (Fig. 9c). Both TSPO and Iba-1 expression were upregulated at 24 h after LPS injection.

## Discussion

The aim of this study was to optimize automated radiosynthesis of the TSPO ligand [^18^F]FEPPA and evaluate the use of [^18^F]FEPPA to detect neuroinflammation in vivo. This work provides the first report of regional-specific distribution of TSPO in vivo in the mouse model of systemic LPS challenge. Our results demonstrate [^18^F]FEPPA rapidly distributed throughout various brain regions and reached a steady-state, which enabled detection of higher regional-specific TSPO expression in the brains of mice injected with LPS. These [^18^F]FEPPA PET/MRI imaging findings were confirmed by western blotting and immunostaining, which demonstrated increased expression of TSPO in the brain was accompanied by microglial activation. Furthermore, real-time RT-PCR revealed an altered immune balance in the brains of LPS-injected mice, including increased levels of both pro-inflammatory and anti-inflammatory cytokines.

We adapted the manual synthesis reported by Wilson et al. [27] into a reliable, optimized automated [^18^F]FEPPA radiosynthesis process using the Eckert and Ziegler Modular system. The non-corrected radiochemical yield for the optimized method was 30 ± 2% within 80 min, which is comparable to 39 ± 3% within 34 min [30] and 30 ± 6% within 36 min [41]. Quality control confirmed that the [^18^F]FEPPA product met all current requirements of a radiotracer for human use. The optimized process produces [^18^F]FEPPA with a consistently high radiochemical purity and specific activity. The final products of both manual and automated synthesis were stable, maintaining a radiochemical purity of more than 99% at 6 h after the end of synthesis. The process is also economical, as no disposable components are required. Quality control analysis confirmed the identity, strength, quality and purity of the radiocompound, and the stability of the product is sufficient for both animal and clinical studies.

Mice were systemically injected with LPS (5 mg/kg, *i.p.*) to evaluate the optimized [^18^F]FEPPA for in vivo PET/MR imaging. After administration of LPS, visualization of [^18^F]FEPPA revealed the distribution and expression of TSPO globally increased in the whole body and brain of the mice, in agreement with previous mouse and rat studies [27, 30, 41]. Our results also demonstrated that [^18^F]FEPPA is rapidly accumulated in the heart, lung and spleen tissues of C57BL6 mice and excreted by the kidneys. PET/MR imaging of [^18^F]FEPPA in LPS-injected mice indicated high specific binding of the radiolabel to TSPO. Higher radioactive signals were observed in the lung, heart and kidney, which express high levels of TSPO under normal conditions. Furthermore, significant upregulation of TSPO was observed in response to induction of inflammation in the peripheral and central organs in the murine LPS model.

LPS-induced animal models have been widely used to research peripheral inflammation or neuroinflammation in order to identify early diagnosis or treatment/prevention approaches [42, 43]. LPS-induced heart injury (10 mg/kg, single *i.p.* injection) leads to morphological changes in the myocardium and increases the levels of creatine kinase and lactate dehydrogenase [44]. Injection of CD14-deficient (CD14-D) mice (25 mg/kg, single injection, *i.p*.) demonstrated that CD14 mediates the LPS-induced proinflammatory responses in the heart and is required for the development of left ventricular dysfunction during LPS-induced shock [45].

However, only limited preclinical data have been reported on in vivo imaging of TSPO in the peripheral tissues. The biodistribution of [^11^C]PK-11195 PET was first reported in canine and human hearts [46] and [^123^I]-Iodo-PK11195 SPECT was evaluated in rat hearts [47]. [^18^F]FEDAC PET was used to assess the expression of TSPO (*B*_max_) in vivo and determine the KD of [^18^F]FEDAC in peripheral tissues; however, [^18^F]FEDAC exhibited relatively low signal-to-noise ratio in peripheral tissues such as the heart and lung [48]. [^18^F]FPBMP was subsequently reported, but leads to weak specific signals for TSPO [49]. Thus, a quantitative, non-invasive and reliable imaging biomarker for TSPO could enable larger, more reliable preclinical and clinical studies.

Cardiovascular or lung imaging in preclinical studies can be complicated by low scanning efficiency, motion correction for the heart or lungs, and partial volume corrections of radioactivity. The resolution of the PET scanner is also a key limitation. However, PET imaging enables assessment of biological disease processes in the heart or lungs, whereas magnetic resonance (MR) scanning provides detailed anatomic imaging and tissue characterization. In this study, advanced 7 T PET/MR imaging was used to identify the heart muscle and detect significant [^18^F]FEPPA uptake in the heart muscle 24 h after LPS challenge compared to controls (Table 1, *p* = 0.05).

In vivo imaging of TSPO in the peripheral tissues can also help to detect inflammation, such as pneumonia [50, 51]. We demonstrated significant [^18^F]FEPPA uptake in the lungs of LPS-injected mice (Table 1, *p* = 0.01). Much lower [^18^F]FEPPA uptake was observed in the liver and blood, which provided a suitable signal-to-noise ratio for peripheral organs such as the heart muscle and lungs. Hatori et al. evaluated the TSPO radioligand *N*-benzyl-*N*-methyl-2-[7,8-dihydro-7-(2-[^18^F]fluoroethyl)-8-oxo-2-phenyl-9*H*-purin-9yl]acetamide ([^18^F]FEDAC) in an acute model of lung injury induced by LPS. In agreement with this study, a similar TAC pattern and clear background were observed in the lungs at 24 h after LPS treatment [52].

Compared to existing non-specific clinical methods such as chest radiography and computed tomography, PET imaging using a TSPO-specific radioligand could potentially provide quantitative information on macrophage trafficking and kinetics in lung diseases, and thus help to evaluate treatment responses and improve our understanding of the pathophysiology of non-infectious inflammatory processes in the lung [53]. Non-invasive PET imaging also enables longitudinal assessments [54], whereas repeat measurements using other invasive methods (e.g., lung biopsy and bronchoalveolar lavage) are unacceptable. The in vivo profiles for [^18^F]FEPPA reached a steady state in the heart muscle and lungs 10 min after injection of [^18^F]FEPPA, and then gradually decreased (Fig. 2). On the one hand, this results demonstrated that [^18^F]FEPPA significantly increased in peripheral organs or tissues (~ 3 folds in heart and lung) in the LPS-inducted group as compared to control mice; but on other hand, the fast metabolic rate in plasma (60% metabolite in 30 min imaging time-window) [30] could increase the difficulty of the quantification accuracy of PET imaging and limit its application for the peripheral inflammation.

The brain exhibited relatively lower [^18^F]FEPPA uptake compared to peripheral organs such as the heart, lung or kidney. Western blotting, real-time PCR and IHC analyses of brain tissue sections for TSPO confirmed the PET/MR imaging detection of significantly higher accumulation of [^18^F]FEPPA in the brains of mice injected with LPS. To investigate whether the specific binding of [^18^F]FEPPA in the brain was due to LPS-induced microglial activation, we used western blotting to detect increased co-expression of TSPO and Iba-1 at 24 h after injection of LPS (Fig. 8, *p* = 0.01 and *p* = 0.05, respectively).

Regional-specific accumulation of [^18^F]FEPPA in the brain reflected the changes in TSPO expression. TSPO ligands have been reported to exert beneficial effects as anti-inflammatory drugs in experimental models of various neurodegenerative diseases and anxiety disorders [55]. More than a dozen anti-inflammatory drugs have been developed [55, 56]. However, the in vivo pharmacokinetic and pharmacodynamics of those anti-inflammatory drugs in specific regions of the brain remain unclear, due to the lack of quantitative or repeatable imaging agents, as described above. In the current study, we used PET/MR-based image segmentation to calculate regional-specific [^18^F]FEPPA accumulation. At 24 h after injection of LPS, the PET SUV images revealed significant increases in [^18^F]FEPPA uptake in the majority of brain regions assessed (Fig. 5a). A pharmacokinetic parameter, total distribution volume VT, revealed a significant increase in [^18^F]FEPPA distribution in the various brain regions of LPS-injected mice compared to control mice (Fig. 5b). Moreover, the AUC_0–30_ min value for [^18^F]FEPPA increased by an average of 1.3-fold compared to the controls (Fig. 5c). These results are in good agreement with previous publications [30].

LPS in the systemic circulation could induce effects in the CNS by crossing the blood–brain barrier (BBB) and directly activating cells within the CNS. The critical issue of whether LPS actually disrupts the BBB and results in over-estimation of [^18^F]FEPPA accumulation in the brain was addressed by Vignal et al. [30]. Our finding also was in a good agreement that VT represents the most robust parameter for quantifying [^18^F]FEPPA uptake and is not affected by changes in cerebral blood flow [28].

The poor correlation between the PET signal intensity and the LPS dose with TSPO imaging agents in rodent models is also important to consider. Therefore, we performed a LPS dose–response study investigate the effectiveness of [^18^F]FEPPA. There was no significant difference in brain [^18^F]FEPPA uptake when the dose of LPS was ≦ 2.5 mg/kg, but significantly higher uptake was observed at 5 mg/kg LPS (Fig. 8). Although the levels of cytokines such as TNF-α, IL-1β andIL-6 in the brain were significantly increased by LPS in a dose-dependent manner (Fig. 8, *p* < 0.005–0.001 at 2.5 mg/kg LPS, *p* < 0.001 at 5 mg/kg LPS), these changes could be explained by two factors. A low dose of LPS may cause a certain degree of BBB disruption [57] or systematic daily administration of LPS (0.5 mg/kg consecutively for 6 days) increased expression of TSPO [58] but this may not significantly affect [^18^F]FEPPA uptake in the brain. Alternatively, this result may indicate the inability of [^18^F]FEPPA to effectively detect the inflammatory responses induced by low doses of LPS. However, several study conditions should be considered, including the strain of LPS, the mouse strain, the dose, site of injection and time course of LPS administration (acute/single or chronic/multiple injections and duration of treatment), the imaging time-points, sensitivity of the PET detector and the specific activity of the PET agents. PET radioligands that target the TSPO have showed promise in the detection of neuroinflammation; however, their preclinical/clinical utility has been hampered by the presence of sensitivity of increased TSPO level. Further studies such as coupling imaging of PET [^18^F]FEPPA and dynamic contrast-enhanced MRI to assess dose-dependence of LPS on BBB permeability/disruption in rodent model.

Accumulation of radioactivity in the brain or peripheral tissues can be due to the radioligand and its radiometabolite(s). However, the stability or metabolism of the radioligand was not tested using blood or tissue samples in this study. In the metabolite assay reported by Vignal et al., approximately 95% of the parent fraction of unmetabolized [^18^F]FEPPA in the brain was identified as the intact form at 30 min after injection [30]. Therefore, at the imaging time-points 0–30 min after injection, the majority of [^18^F]FEPPA bound to TSPO in the brain, heart, lung and kidney is likely to be due to [^18^F] FEPPA.

The presence of TSPO in the brain was confirmed by Western blotting. In comparison to controls, TSPO and the microglial marker Iba-1 were expressed at significantly higher levels in the brain at 24 h after LPS injection (Fig. 9c, *P* < *0.05*). Based on IHC, cerebral TSPO expression colocalized with Iba-1 (a microglial marker) in LPS-injected mice, consistent with previous findings [59, 60]. Thus, TSPO represents a suitable molecular target to modulate microglia/macrophage function during neuroinflammation. Moreover, TSPO expression was assessed in various brain tissues. Quantitative analysis of immunohistological staining demonstrated that TSPO was significantly upregulated in the CA1, CA3 and cerebellum by LPS (Fig. 9).

## Conclusions

The second-generation TSPO radioligand [^18^F]FEPPA has been extensively used in clinical or preclinical development to study neuroinflammation and peripheral-type inflammation. We optimized an automated radiosynthesis method on the Eckert & Ziegler Modular system that meets cGMP requirements and produces [^18^F]FEPPA at high purity and high yield. The experimental evidence in this study indicates [^18^F]FEPPA can be employed to monitor moderate-to-severe neuroinflammatory processes in vivo using PET. [^18^F]FEPPA uptake/binding were quantified in various anatomical brain regions, and ligand detected significant increased TSPO levels in the brain of systemic LPS-induced neuroinflammation (5 mg/kg). More studies are needed to determine the sensitivity of [^18^F]FEPPA as an imaging biomarker of chronic or mild neuroinflammation and then future explore its capacity to differentiate the severity of neuroinflammation in animal or human studies on TSPO-related neuroinflammation.

## Supplementary Information

**Additional file 1. Table 1**: List of reagents used in the automated procedure.

**Additional file 2. Fig. 1**: [^18^F]FEPPA radiosynthesis on the Eckert and Ziegler modular system. [^18^F]FEPPA was synthesized using an in-house reaction sequence; the reagents used in the automated procedure are listed in Additional file 1: Table 1.

**Additional file 3. Fig. 2**: Time activity curves for [^18^F]FEPPA radioactivity (SUV) in blood, heart muscle, liver, lung, spleen and kidney. This figure is based on same data as Fig. 1, but over a different time scale (0‒3 min). Data are mean ± SEM of 6 mice per group.

**Additional file 4. Fig. 3**: Time-activity curves for animal PET/MR studies of [^18^F]FEPPA of the brain for the control group and 24 h after LPS injection (5 mg/kg). The same data source from Fig. 1 but with different time scale (0–3 min). Data are mean ± SEM of 6 mice per group.

## Data Availability

The datasets used and/or analyzed during the current study are available from the corresponding author on reasonable request.

## Abbreviations

7 T PET/MR: 7-T Positron emission tomography/ Magnetic resonance imaging
[^18^F]FEPPA: [18F]-*N*-(2-(2-Fluoroethoxy)benzyl)-*N*-(4-phenoxypyridin-3-yl)
AUC: Area under curve
CK: Creatine kinase
CNS: Central nervous system
Il-1β: Interleukin 1 beta
Il-6: Interleukin-6
LDH: Lactate dehydrogenase
LPS: Lipopolysaccharides
PBR: Peripheral-type benzodiazepine receptor
RT-PCT: Real-time reverse transcription-polymerase chain reaction
SEM: Standard error of the mean
SPECT: Single-photon emission computed tomography
SUV: Standard uptake value
TAC: Time-activity curve
TNF-α: Tumor necrosis factor Alpha
TSPO: Translocator protein 18 ka
VT: Total volume of distribution
