# Supplementary material for: GMP-compliant fully automated radiosynthesis of [18F]FEPPA for PET/MRI imaging of regional brain TSPO expression
Source: EJNMMI Res. 2021 Mar 16;11:26. doi: 10.1186/s13550-021-00768-9 (PMC7966678; doi:10.1186/s13550-021-00768-9)
Supplement: Supplementary file 1 — Additional file 1. Table 1: List of reagents used in the automated procedure. [file 13550_2021_768_MOESM1_ESM.docx]

**Supplementary Table**

**Suppl.Table 1:** List of reagents used in the automated procedure.

| **Vial number** | **Reagents** | **Volumes** |
| --- | --- | --- |
| vial A | Recovered H_2_^18^O |  |
| vial B | Eluent QMA (K_2_CO_3_/K222 in CH_3_CN/H_2_O, 44/11, v/v) | 1 mL |
| vial C | CH_3_CN anhydrous | 0.8 mL |
| vial D | CH_3_CN anhydrous | 0.8 mL |
| vial E | Mobile phase (1/1 CH_3_CN /H_2_O + 0.5% phosphoric acid) | 2 mL |
| vial F | Precursor | 5 mg |
| vial G | Collect reaction mixture |  |
| vial H | H_2_O (to mix with the collected LC fraction) | 40 mL |
| vial I | H_2_O (to remove CH3CN) | 15 mL |
| vial J | Ethanol | 0.9 mL |
| vial K | Normal saline | 5 mL |
| vial L | sterile vial containing 5 ml saline (for collecting product) | |
| vial M | Mobile phase (1/1 CH_3_CN /H_2_O + 0.5% phosphoric acid) |  |
|  |  |  |
